# Global trends in research of immune cells associated with hypertensive disorders of pregnancy: A 20-year bibliometric analyses (from 2001 to 2021)

**DOI:** 10.3389/fimmu.2022.1036461

**Published:** 2023-01-09

**Authors:** Yue Wang, Baoxuan Li, Fei Tong

**Affiliations:** ^1^ Department of Obstetrics and Gynaecology, Shengjing Hospital of China Medical University, Shenyang, China; ^2^ Department of Cardiology, Shengjing Hospital of China Medical University, Shenyang, China

**Keywords:** hypertensive disorders of pregnancy (HDP), immune cells, bibliometric analysis, inflammation, research hotspots

## Abstract

**Background:**

A growing evidence suggests that immune cells play a significant role in the pathogenesis of hypertensive disorders of pregnancy (HDP).Over the past 20 years, several studies have been conducted on the role of immune cells in hypertensive disorders of pregnancy. This study used bibliometric analysis to assess research hotspots and future trends in studies on immune cells in hypertensive disorders of pregnancy.

**Methods:**

We extracted all relevant literature on immune cells and hypertensive disorders of pregnancy from the Web of Science core collection for the period of 2001 to 2021. We used VOS Viewer, CiteSpace, R-bibliometrix and Python for bibliometric analysis.

**Results:**

We identified 2,388 records published in 593 journals by 9,886 authors from 2,174 universities/institutions in 91 countries/regions. The number of publications tended to increase over time, with the highest number of publications in 2021, up to 205. The USA was the country with the most publications. UNIVERSITY OF MISSISSIPPI was the most influential institution. Lamarca B, Romero R, and Saito S were the most prolific authors. Finally, three research hotspot clusters were identified based on keywords, which reflected the role of immune cells in the development of hypertensive disorders of pregnancy, the current research status,and predicted hot spots for future research.

**Conclusions:**

Our study systematically analyzed the role of immune cells in the pathogenesis of hypertensive disorders of pregnancy in the last 20 years. Our results indicated that immune cells, such as T cells, natural killer (NK) cells,and macrophages, and the cytokines released such as TNF-α, IFN-γ in the maternal circulation and at the maternal-fetal interface would influence the development of hypertensive disorders of pregnancy and we need further investigate the role of individual immune cells and translational studies to provide new therapeutic perspectives to mitigate adverse perinatal outcomes due to hypertensive disorders of pregnancy. In conclusion, bibliometric studies provide a general overview of immune cells in the study of hypertensive disorders of pregnancy.

## Introduction

Hypertensive disorders of pregnancy (HDP) mainly include chronic hypertension (diagnosed before 20-weeks of pregnancy), new-onset hypertension (either preeclampsia or gestational hypertension, diagnosed after 20-weeks of pregnancy) (1).Despite some differences in the level of medical care in different countries/regions, HDP remain a common cause for severe threat to maternal-child health and life, even in developed countries.The statistics from 2003 to 2009 showed that HDP were responsible for 14% of maternal deaths worldwide and the second leading direct cause of maternal death following haemorrhage (2). HDP, especially preeclampsia, cause significant healthcare expenditures globally (3).It has been estimated that in 2012, $2.18 billion was spent on health care during 12 months after delivery in the United States for patients with preeclampsia (4).In addition, the incidence of HDP is rapidly increasing.The statistics found a 25% increase in the incidence of preeclampsia in the United States between 1987 and 2004 (5). Even after delivery, the adverse effects of HDP(especially preeclampsia) persist, and studies have found that it increased the risk of long-term cardiovascular and metabolic disease in the mother and offspring (6). HDP has been a major concern for obstetricians worldwide due to the high incidence of adverse pregnancy outcomes, and high health care costs. It is well established that both gestational hypertension and pregnancy with chronic hypertension have a tendency to progress to preeclampsia, which results in a high maternal and fetal mortality. The pathophysiology is complex and heterogeneous, and the exact etiology is still unknown, however, abnormal placental development in early pregnancy and subsequent systemic endothelial dysfunction are two currently recognized stages in the development of preeclampsia (7). Studies have shown that the balance of immune cells and the factors they produce are essential for the normal pregnancy and any imbalance may induce the impaired trophoblast invasion, causing insufficient maternal spiral artery remodeling, and eventually abnormal placental development and placental hypoxia. Therefore, it is important to analyze the research history of immune cells and HDP development, the current research status and future scientific prospects.

The field of HDP development related to immune cells has undergone numerous researches and made great progress in the last 20 years, however, there is a lack of global comprehensive reports that could be beneficial for researchers to gain insight into the progress of research and to find global reports with future research trends. Bibliometrics, based on the application of mathematical and statistical methods, allows quantitative analysis and summary visualization of data from published publications in a specific research area (8, 9). The obtained result data or information gives access to the comprehension of the history and the current state of scientific research achievements in the field, and the prediction of future research directions and opportunities for collaboration (10, 11). Currently, no study has focused on the research hotspots, research collaborations worldwide and future trends in the field of HDP development and immune cells. In this study, we analyzed the researches in the field of HDP development and immune cells related to the last 20 years by virtue of bibliometric methods, combined with clustering research, to analyze the historical evolution of researches in the field, related hotspots, and to gain insight into the research trends in the field.

## Materials and methods

### Data collection

Data was downloaded on Jane 3, 2022 for a search of the Web of Science Core Collection (WoSCC: SCI-E and SSCI). Compared to other databases,the WoSCC is more accurate. In addition, WoSCC contains almost all influential and high quality journals which can be downloaded to a comprehensive citation index record (12).We set the following search strategy:TS=[(pregnancy-induced hypertension OR pregnancy induced hypertension OR gestational hypertension OR chronic hypertension with superimposed preeclampsia OR eclampsia OR preeclampsia OR hypertensive disorder complicating pregnancy OR pregnancy hypertension OR hdct OR hypertension of pregnancy OR pregnancy with chronic hypertension OR pregnancy-associated hypertension OR pregnancy associated hypertension OR hypertensive disorders of pregnancy OR EPH-gestosis OR “pregnancy toxemia”) AND ”immune cell*” OR “macrophage*” OR “mast cell*” OR “dendritic cell*” OR “NK cell*” OR “natural killer cell*” OR “lymphocyte*” OR “T cell*” OR “B cell*” OR “migDC*” OR “MAIT cell*” OR “plasmablast*” OR “ILC*”OR “monocyte*” OR “mononuclear phagocyte*” OR “lymphoid cell*” OR “plasma cell*” OR “eosinophil*” OR “neutrophil*” OR “treg*” OR “T-helper” OR “T helper” OR “Th cell*” OR Th1 OR Th2 OR Th3 OR Th9 OR Th16 OR Th17 OR Th22 OR Tfh OR DC)].

The search for this study was limited to the period of 2001 to 2021, restricted to English, and limited to article or review. Data collected included title, author, institution, country, abstract, journal, keywords, and references. All literature searched and data downloaded were done on the same day to avoid bias after database updates. Two investigators (YW and BL) were screened independently with 95% agreement (kappa=(P0-Pe)/(n-Pe)=0.95>0.75), consistent with a significant agreement of results. In addition, all data were obtained from public databases and informed consent was not required.

### Data analysis and visualization

In this study, detailed records were provided in WoSCC, including author, country, journal, institution, publication time, impact factor (IF), and Hirsch index (H-index). The impact factor (IF) of the relevant journal was obtained by the 2020 Journal Citation Reports (JCR), which is an important indicator for assessing the scientific value of research. H-index is defined as the number of papers published by a scholar, journal, or country/region with h papers with ≥ h citations, which is an important indicator to assess the scholarly impact of a scholar, journal, or country/region (13). Python 3.8.2 was used to visualize international maps of publications in different countries/regions. VOSviewer 1.6.18 (Leiden University, Leiden, The Netherlands) was used to analyze bibliometric networks of authors, institutions, journals, and keywords and to generate visual maps (14).The online analysis platform (http://bibliometric.com/) was used to analyze and visualize international collaboration between countries. Microsoft Power BI was applied to visualize the number of publications and citations/regions. CiteSpace 6.1.2 was provided to extract highly cited publications and generate visual map. In addition, R–bibliometrix was utilized for keyword extraction and historical citation graph analysis.

## Results

### Search results

**Figure 1:**
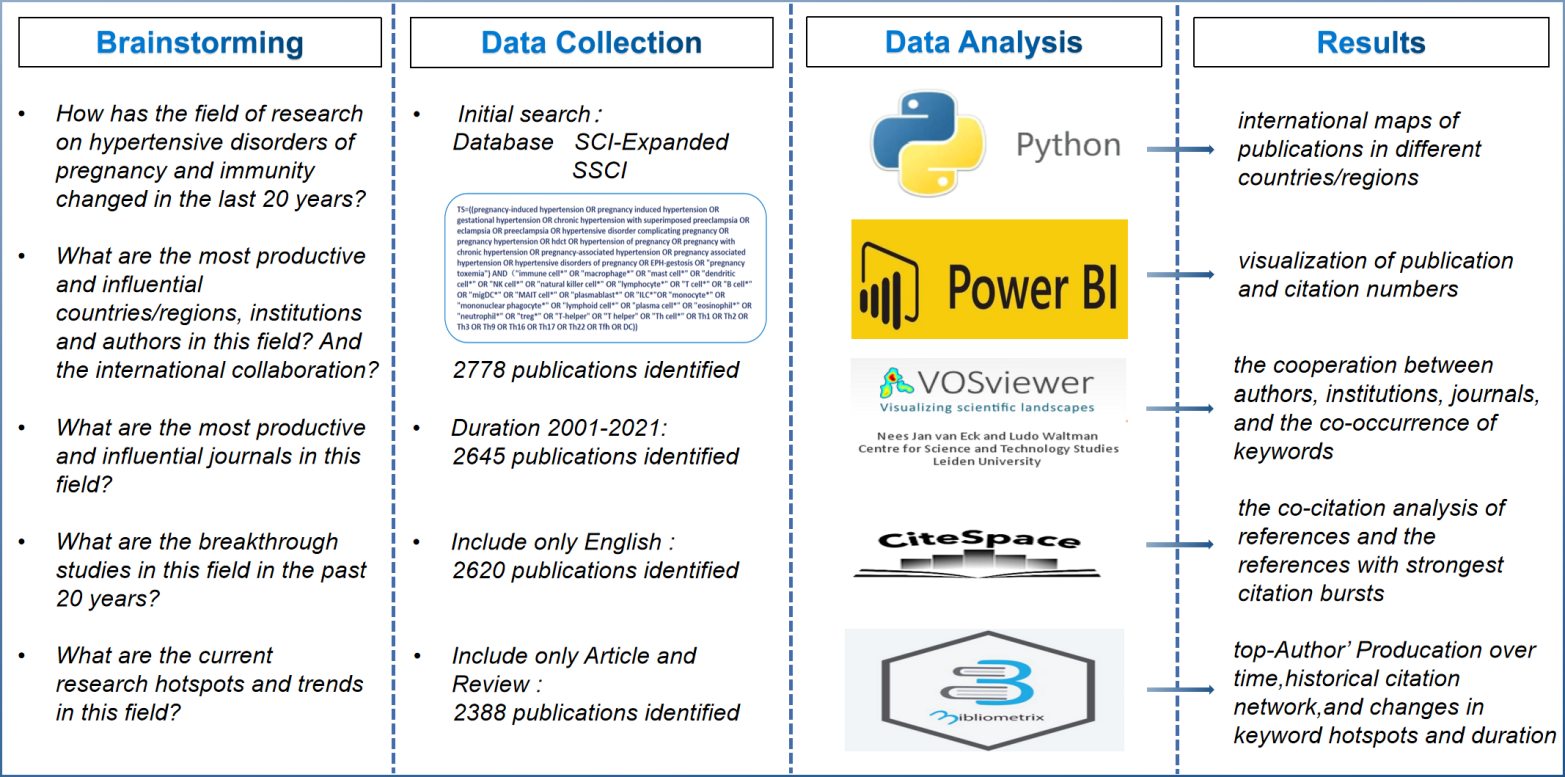
Conceptual design of the entire study.

A total of 2,778 publications from Science Citation Index Expanded (SCI-Expanded), Social Sciences Citation Index (SSCI) were screened from WoSCC through the Chinese Medical University Library website. The search period for restricting this study was 2001 to 2021, and a total of 2,645 publications related to HDP and immune cells were retrieved. We further restricted the language to English, and the article type excluded non-article and non-review publications. Finally, a total of 2,388 publications (1,938 articles and 450 reviews) could be included in the bibliometric analysis. The conceptual design of the entire study is shown in **Figure 1**.

### Annual growth trend in publications and citations

**Figure 2:**
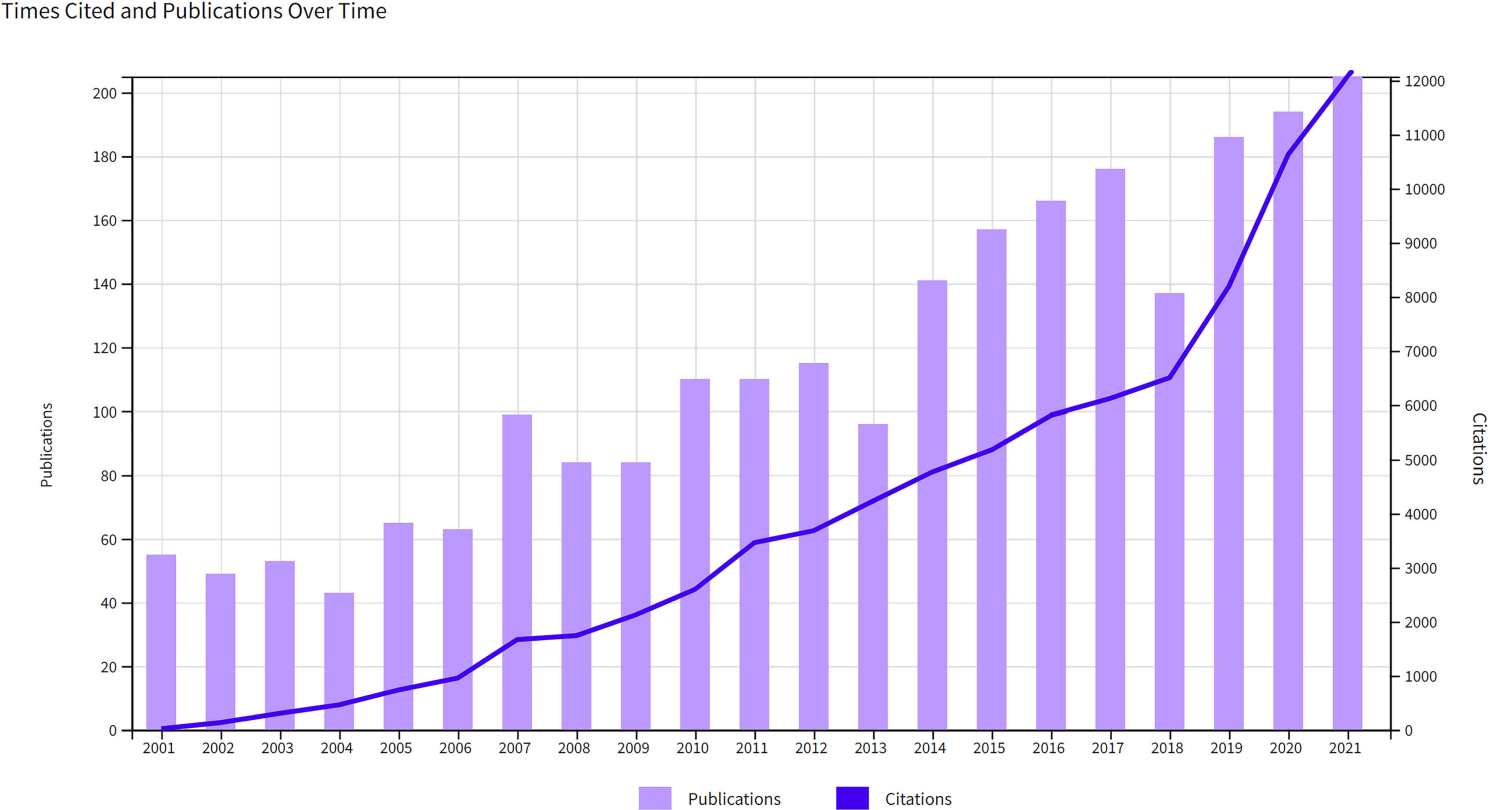
Annual publications and citations growth curve from 2001 to 2021.

There was an increasing trend in the number of publications and citations per year (**Figure 2**). During the study period, the number increased significantly from 55 in 2001 to 205 in 2021. 2,388 publications were cited 85,578 times, with an average of 35.84 citations per paper and an H-index of 120, and the number of citations increased significantly from 19 in 2001 to 12,072 in 2021. These dramatic increases in publications and annual citations indicated a surge of interest in the study of the immune cells associated with HPD in recent years.

### Contributions of countries/regions

**Figure 3:**
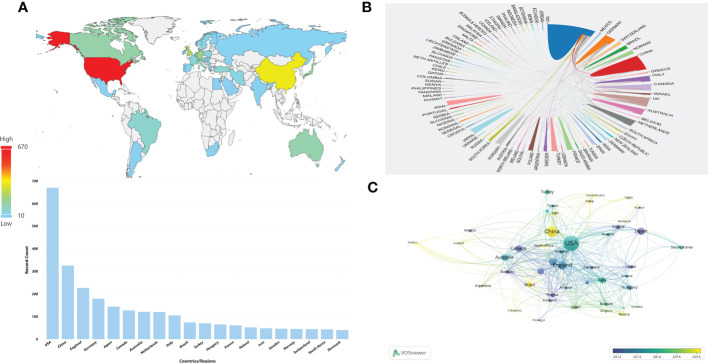
Distribution of countries or regions and mutual cooperation relations. **(A)** Distribution of the top 10 countries/regions in terms of number of publications on the world map. **(B)** The cooperative network map between countries or regions. The color represents different countries/regions,the area represents the number of citations in each country or region, and the thickness of the connecting line indicates the cooperation frequency. **(C)** Overlay visualization of country/region cooperation analysis. The circles indicate different countries/regions, the color represents the average year, the area of the circle represents the number of citations in each country or region, the distance indicates the correlation between each country/region, and the thickness of the connecting line indicates the strength of co-authorship cooperation.

A total of 91 countries/regions provided publications on HPD and immune cells between 2001 and 2021, with a total of 43 countries/regions having more than 10 publications, as shown in the world map in **Figure 3A**. The USA (n = 670) was the most productive country with more than a quarter of the included publications, followed by CHINA (n = 325), ENGLAND (n = 227), GERMANY (n = 179), and JAPAN (n = 144) (**Table 1**). The network map of cooperation between countries or regions showed that the USA was the country with the highest number of articles and the most active international cooperation(**Figure 3B**).As shown in **Figure 3C**, there was close cooperation between different countries or regions in the world. When we set the minimum number of publications and citations of a country/region to 5, a total of 45 countries/regions met the requirements, and the analysis of international cooperation revealed that the USA was involved in the highest frequency of international cooperation. China has conducted relevant researches with several countries in the recent years.

**Table 1:** The top 20 most productive journals published in HDP and immune cells research.

Rank	Countries/Regions	Record Count	% of 2,388
1	USA	670	28.057
2	China	325	13.61
3	England	227	9.506
4	Germany	179	7.496
5	Japan	144	6.03
6	Canada	127	5.318
7	Australia	121	5.067
8	Netherlands	120	5.025
9	Italy	105	4.397
10	Brazil	74	3.099
11	Turkey	70	2.931
12	Hungary	65	2.722
13	France	61	2.554
14	Poland	52	2.178
15	Iran	48	2.01
16	Sweden	46	1.926
17	Norway	45	1.884
18	Switzerland	44	1.843
19	South Korea	43	1.801
20	Denmark	40	1.675

### Analysis of institutional output

**Figure 4:**
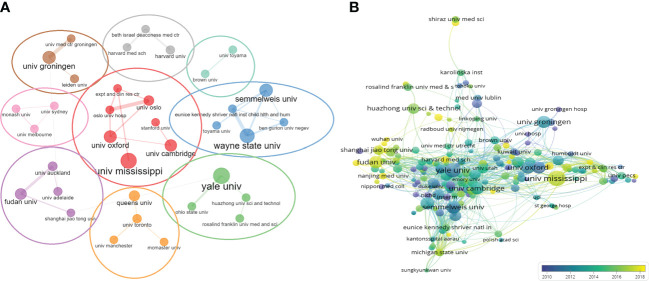
Network map of universities/institutions collaboration. **(A)** The network visualization of universities/institutions (The thickness of the lines indicates the strength of cooperation between universities/institutions). **(B)** Dynamics and trends of universities/institutions over time.

We then evaluated the number of publications from 2,174 universities or institutions worldwide. UNIVERSITY OF MISSISSIPPI, with a total of 66 publications, had the highest number of publications in the last 20 years, followed by YALE UNIVERSITY (n = 61), UNIVERSITY OF LONDON (n = 52). We analyzed the collaborative relationships between universities or institutions. Among the top 50 universities or institutions, 48 have established inter-collaborative relationships, and further visualization was shown in **Figure 4A**. It could form nine clusters, respectively, with UNIVERSITY OF MISSISSIPPI (univ mississippi), YALE UNIVERSITY (yale univ),Queen’s University (queens univ),Fudan University (fudan univ),UNIVERSITY OF SYDNEY(univ sydney),University of Groningen (univ groningen),HARVARD UNIVERSITY(harvard univ),Brown University (brown univ),WAYNE STATE UNIVERSITY(wayne state univ) as the main sponsoring research universities or institutions. UNIVERSITY OF MISSISSIPPI (n = 66) was the largest sponsoring institution, with close cooperation between several institutions. Subsequently, we applied VOSviewer software to construct a collaborative network, setting the threshold to a minimum number of publications of 5 and a minimum number of citations of 100, and identified a total of 208 universities or institutions. As shown in **Figure 4B**, the collaboration among universities or institutions gradually increased after 2010 over time. UNIVERSITY OF MISSISSIPPI was the most active institution in international collaboration in the past 5 years, and those in China such as Fudan University, Nanjing Medical University and Wuhan University had produced more studies and were active in international collaboration in the past 3 years.

### Analysis of authorship

**Figure 5:**
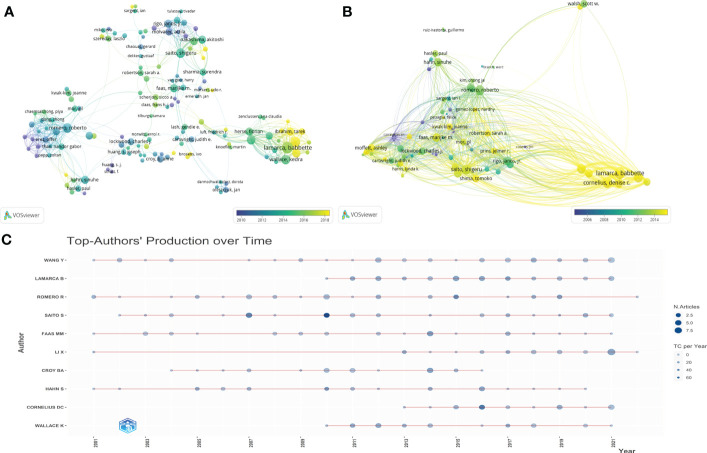
Authorship and co-authorship and citation analysis in the field of HDP and immune cells. **(A)** Network visualization of author co-authorship. **(B)** Network visualization of co-authorship citation counts. **(C)** Top-Author’ Producation over time.

We analyzed 10,758 authors worldwide who have contributed to the field of HPD and immune cells. The top 10 authors in the research area are listed in **Table 2**, where Lamarca B from the University of Mississippi, USA, ranked first in terms of publications (n=40) and second in the number of citations, followed by Romero R from Wayne State University, USA, was the second author in publication (n=37).In addition, Saito S from the University of Toyama, Japan, was the third author in publication (n=35), howerver, was the first author who had the highest total number of citations. By the minimum number of documents of an anthor set to 5 and the minimum number of citations of an anthor set to 100, 142 authors who met the threshold were identified (**Figure 5A**). We then set the minimum number of citations of an author threshold to 500 and finally identified 80 authors who met the threshold (**Figure 5B**). As shown in **Figure 5A**, we found five collaborative teams actively involved in the field of HPD and immune cells,and Lamarca B and Ibrahim T as authors who have made significant contributions to this research field in the last 5 years.The articles of Lamarca B have been widely cited in recent years. As shown in **Figure 5C**, the publication record of top authors over time showed that FAAS MM had been working on HPD and immune cells since 2001 and still had new articles published until 2021.LAMARCA B has been publishing articles related to HPD and immune cells since 2010 and has been publishing new articles every year and has become the author with the highest number of published articles in the field. LAMARCA B, FAAS MM, CORNELIUS DC, LI X, SAITO S and WANG Y were the most published authors in this field with 3 articles in the last 3 years.

**Table 2:** The top 10 most productive authors in HDP and immune cells research.

Rank	Author	Article counts	Total number of citations	Average number of citations	First author counts	First author citation counts	Corresponding author counts	Corresponding author citation counts
1	Lamarca B	40	557	13.93	6	111	33	500
2	Romero R	37	359	9.7	1	0	0	18
3	Saito S	35	891	25.46	8	364	45.5	22
4	Cornelius DC	32	280	8.75	5	85	17	7
5	Faas MM	32	278	8.69	11	121	11	17
6	Wallace K	29	454	15.66	5	94	18.8	9
7	Dechend R	29	352	12.14	0	0	0	5
8	Rigo J	25	440	17.6	0	0	0	0
9	Hahn S	25	306	12.24	3	29	9.67	12
10	Moffett A	24	523	21.79	4	59	14.75	11

### Journal distribution

In this study, a total of 593 journals were involved in the publication of articles related to HDP and immune cells, and a comprehensive analysis of these journals was performed, including journal name, number of articles, total citations, citations per article, IF (2020), Quartile in category and H-index. **Table 3** shows the 10 most influential journals in this research area; these journals published 806 publications, accounting for 33.75% of the total publication volume. AMERICAN JOURNAL OF REPRODUCTIVE IMMUNOLOGY (n=167), JOURNAL OF REPRODUCTIVE IMMUNOLOGY (n=146) and PLACENTA (n=119) were the top three journals in the number of relevant articles published. They were also the top three journals in terms of total citations (1,550, 1,366 and 796, respectively). The top three journals with the highest average number of citations per paper were LANCET, JOURNAL OF EXPERIMENTAL MEDICINE and ANNUAL REVIEW OF IMMUNOLOGY(85.00, 74.67 and 60.00).HYPERTENSION, JOURNAL OF REPRODUCTIVE IMMUNOLOGY and AMERICAN JOURNAL OF OBSTETRICS AND GYNECOLOGY had the highest IF (2020) among the top 10 journals in terms of the number of publications (10.86 vs 9.36 vs 7.561, respectively).

**Table 3:** The top 10 most productive journals published in HDP and immune cells research.

Rank	Journal title	Article counts	Total number of citations	Average number of citations	IF (2020)	Quartile in category (2020)	H-index
1	AMERICAN JOURNAL OF REPRODUCTIVE IMMUNOLOGY	167	1550	9.28	3.886	Q3	97
2	JOURNAL OF REPRODUCTIVE IMMUNOLOGY	146	1366	9.36	4.054	Q3	87
3	PLACENTA	119	796	6.69	3.481	Q1	124
4	FRONTIERS IN IMMUNOLOGY	77	376	4.88	7.561	Q2	124
5	AMERICAN JOURNAL OF OBSTETRICS AND GYNECOLOGY	55	514	9.35	8.661	Q1	225
6	HYPERTENSION IN PREGNANCY	52	352	6.77	2.108	Q4	46
7	PLOS ONE	50	222	4.44	3.24	Q2	332
8	HYPERTENSION	49	532	10.86	10.19	Q1	265
9	JOURNAL OF MATERNAL-FETAL & NEONATAL MEDICINE	46	191	4.15	2.398	Q3	80
10	PREGNANCY HYPERTENSION-AN INTERNATIONAL JOURNAL OF WOMENS CARDIOVASCULAR HEALTH	45	100	2.22	2.899	Q4	17

### Historical evolution of HDP and immune cells research

**Figure 6:**
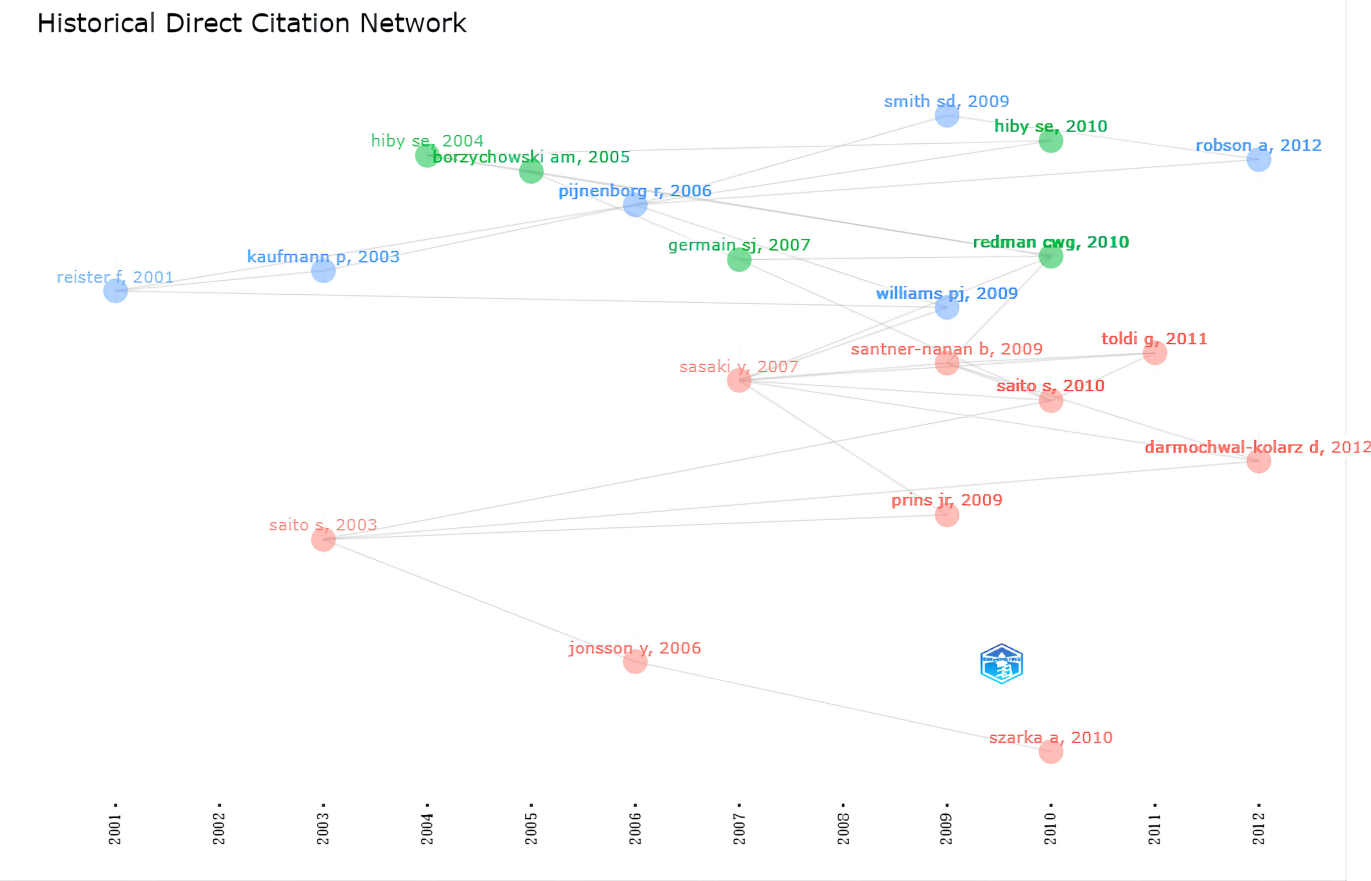
Historiograph of HDP and immune cells research.

We further analyzed the historiograph of HDP and immune cells research in order to understand the publications that were important in the history of the discipline (**Figure 6**), which are shown in **Table 4**, and we found that the significant studies in the field were published from 2001 to 2013. In 2001, Reister F suggested that maternal macrophages induce apoptosis in extravillous trophoblast cells and that apoptosis of extravillous trophoblast cells attracts and activates more macrophages, which is associated with impaired invasion of the uteroplacental artery in preeclampsia. Two influential reviews were published in 2003 on the interaction of immunity with endothelial dysfunction and poor placental formation. In 2004, it was found that the killer immunoglobulin receptor (KIR) on natural killer (NK) cells in the maternal decidua recognizes the polymorphic histocompatibility antigen HLA-C on the fetal trophectoderm as a key factor in the development of preeclampsia, thus proposing that patients with KIR A haploid (KIR AA) are at increased risk of developing preeclampsia. Further studies in 2005 found significant changes in the lymphocytes of the innate immune system, including NK cells and NK T cells, in women with preeclampsia, thus suggesting a major role for the innate immune system in the development of the disease. Based on a review published in 2006, we found that by this time several experiments had highlighted the important role of uterine NK cells in the pathogenesis of preeclampsia. Based on a review published in 2006, we found that by this time several experiments had highlighted the important role of uterine NK cells in the pathogenesis of preeclampsia. In addition, the study found increased levels of IL-6 and IL-8 in the blood of patients with preeclampsia compared to women with normal pregnancies, supporting the hypothesis of an increased inflammatory response in preeclampsia.

**Table 4:** Historiograph of HDP and immune cells research.

Rank	Title	Journal	Year	LCS	GCS
1	Macrophage-induced apoptosis limits endovascular trophoblast invasion in the uterine wall of preeclamptic women	LAB INVEST	2001	66	204
2	Endovascular trophoblast invasion: Implications for the pathogenesis of intrauterine growth retardation and preeclampsia	BIOL REPROD	2003	71	789
3	Th1/Th2 balance in preeclampsia	J REPROD IMMUNOL	2003	101	223
4	Combinations of maternal kir and fetal hla-c genes influence the risk of preeclampsia and reproductive success	J EXP MED	2004	185	775
5	Changes in systemic type 1 and type 2 immunity in normal pregnancy and pre-eclampsia may be mediated by natural killer cells	EUR J IMMUNOL	2005	76	159
6	The uterine spiral arteries in human pregnancy: Facts and controversies	PLACENTA	2006	113	731
7	Cytokine mapping of sera from women with preeclampsia and normal pregnancies	J Reprod Immunol	2006	72	188
8	Systemic inflammatory priming in normal pregnancy and preeclampsia: The role of circulating syncytiotrophoblast microparticles	J IMMUNOL	2007	80	312
9	Proportion of peripheral blood and decidual cd4(+) cd25(Bright) regulatory t cells in pre-eclampsia	CLIN EXP IMMUNOL	2007	133	243
10	Preeclampsia is associated with lower percentages of regulatory t cells in maternal blood	HYPERTENS PREGNANCY	2009	74	109
11	Altered decidual leucocyte populations in the placental bed in pre-eclampsia and foetal growth restriction: A comparison with late normal pregnancy	REPRODUCTION	2009	62	102
12	Evidence for immune cell involvement in decidual spiral arteriole remodeling in early human pregnancy	AM J PATHOL	2009	104	280
13	Systemic increase in the ratio between foxp3(+) and il-17-producing cd4(+) t cells in healthy pregnancy but not in preeclampsia	J IMMUNOL	2009	149	315
14	Maternal activating kirs protect against human reproductive failure mediated by fetal hla-c2	J CLIN INVEST	2010	70	316
15	Immunology of pre-eclampsia	AM J REPROD IMMUNOL	2010	118	443
16	Th1/Th2/Th17 and regulatory t-cell paradigm in pregnancy	AM J REPROD IMMUNOL	2010	124	712
17	Circulating cytokines, chemokines and adhesion molecules in normal pregnancy and preeclampsia determined by multiplex suspension array	BMC Immunol	2010	83	340
18	Increased prevalence of IL-17-producing peripheral blood lymphocytes in pre-eclampsia	AM J REPROD IMMUNOL	2011	67	115
19	The predominance of th17 lymphocytes and decreased number and function of treg cells in preeclampsia	J REPROD IMMUNOL	2012	106	166
20	Uterine natural killer cells initiate spiral artery remodeling in human pregnancy	FASEB J	2012	63	196

GCS, the total number of citations in Web of Science; LCS, the number of citations in the current dataset.

Two new perspectives proposed in 2007 were that circulating syncytiotrophoblast microparticles(STBM) bound to monocyte junctions stimulate inflammatory cytokines as an influential factor in the systemic inflammatory response that induces pre-eclampsia. The reduced number of T regulatory cells (Treg) in patients with preeclampsia may affect maternal tolerance of the fetus. Subsequently, in 2009, it was found that leukocyte populations (NK cells & macrophages) in the uterine decidua have an essential influence on trophoblast invasion defects and spiral artery remodeling in early gestation. In 2010, connecting previous studies, the relevance of the pathogenesis of preeclampsia to immune mechanisms at the maternal-fetal interface was determined for the first time. In addition, the association of preeclampsia with a generalized pro-inflammatory systemic environment has been verified at the level of blood circulation. The central role of activating the combination of KIR on NK cells and HLA-C, and Tregs interaction with indoleamine 2,3-dioxygenase in the development of pathological pregnancy were identified. Changes in immune cells in patients with pre-eclampsia were further explored in 2011 and 2012.This has established the basis for more in-depth studies in recent years on hypertensive disorders of pregnancy and immune cells.

### Mutation detection of co–citation analysis

**Figure 7:**
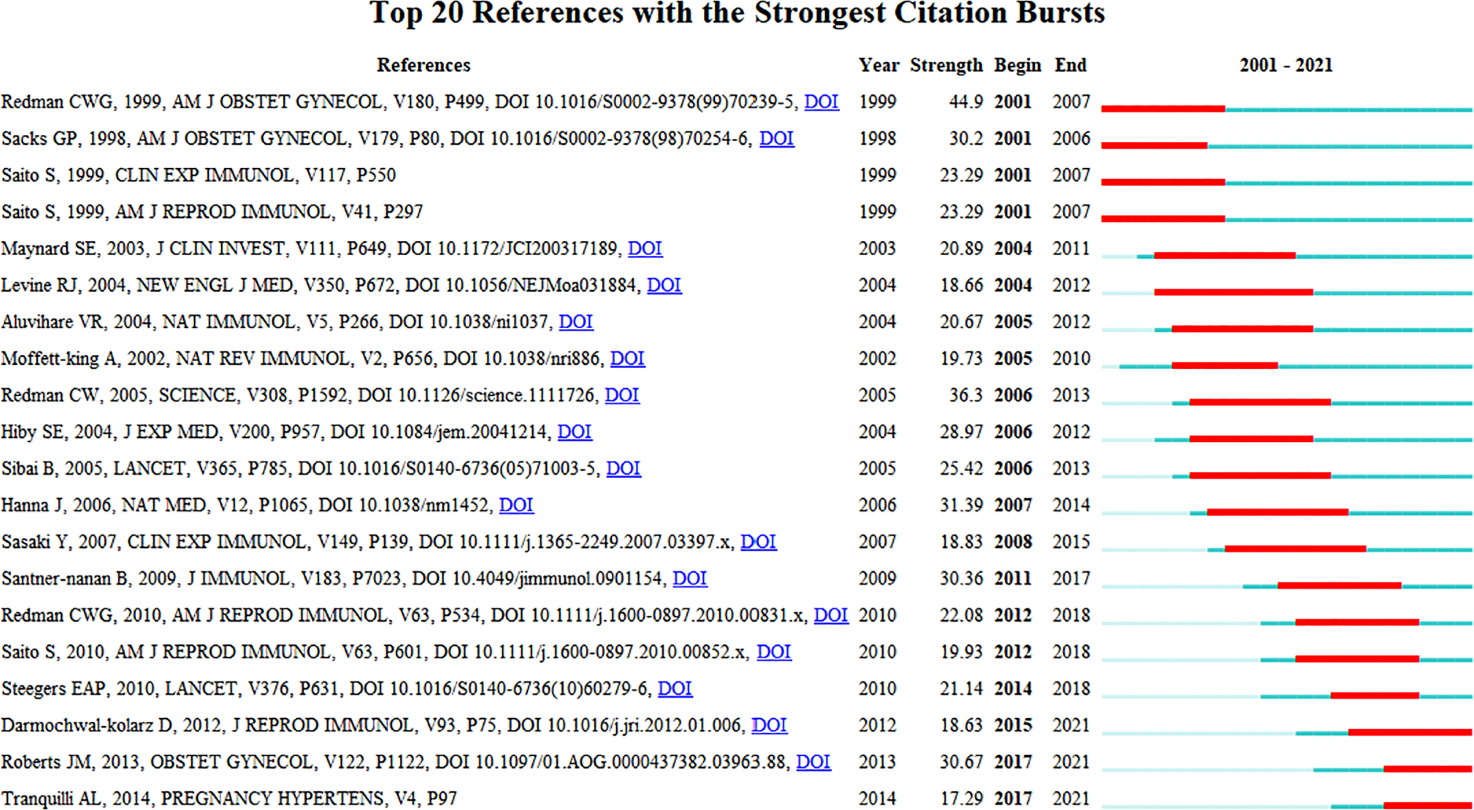
Mutation detection of co–citation analysis (outbreak durations≥4 years).

The co-citation analysis provides insight into past HDP and immune cell research hotspots in the past and predicts future research directions. Setting the duration of the burst at greater than or equal to 4 years, the top 20 most cited articles are listed in **Figure 7**. The peak of article citations tends to be 1 - 2 years after the publication of the article and lasts for 4-5 years. Multiple highly cited articles appeared from 2001 to 2015.In the last 3 years, the heat of highly cited articles continues to the present, and the current research hotspots are immune imbalances, such as increased pro-inflammatory CD4+ T cells, decreased Treg, the Th1/Th2 immune balance, up-regulation Th17 cells, and regulatory cytokine IL-10, affecting the progression of preeclampsia. We anticipate that future research will continue to focus on the impact of different components of the immune system at the maternal-fetal interface on the progression of HDP, and on the development of effective drugs to prevent or improve maternal and fetal outcomes based on the physiological mechanisms of pathogenesis.

### Trends in HDP and immune cells research

**Figure 8:**
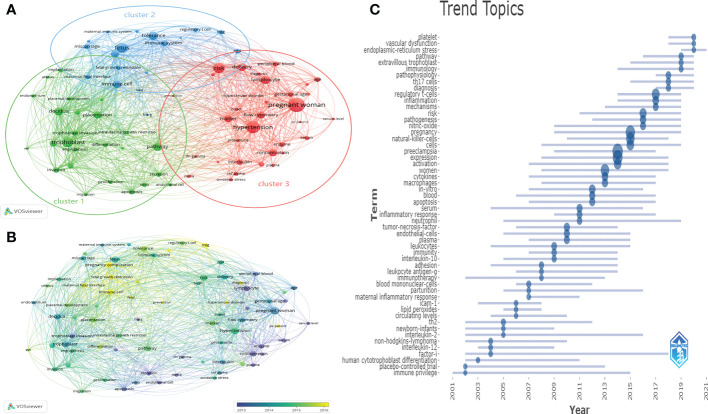
Distribution of keywords (frequency > 50) **(A)** Clustering map of keywords. **(B)** Clustering map of keywords according to the average time of appearance. **(C)** Trend of keywords over time and duration of study.

From the 2,388 published records, we could extract 41,716 keywords. The keywords with more than 100 occurrences were divided into three clusters by co-occurrence analysis, which facilitated a systematic understanding between keywords in the field (**Figure 8A**). The three most frequently used keywords in the three clusters were “pregnant woman”, “trophoblast” and “fetus”. Cluster 1, the study focuses on immune cell changes in the maternal decidua during trophoblast invasion. migration, apoptosis, vitro, endothelial cell, proliferation, secretion, invasion, pathway differentiation, angiogenesis, trophoblast, trophoblast invasion, intrauterine growth restrictio, placentation, decidua, placental development, endometrium, uterus, and implantation are receiving increasing attention. Cluster 2, immune tolerance to semi-isogenic fetal antigens at the maternal-fetal interface is the main focus, including maternal immune system, miscarriage, fetus, fetal growth restriction, maternal fetal interface, immune cell, fetal, hlag, regulatory t cell, treg, cd4, tolerance, immune system. Cluster 3, “prenant woman” is the largest node, which focuses on clinical syndromes that occur in prenant women, with the main focus including oxidative stress, tnf alpha, interleukin, plasma concentration, enzyme, ifn gamma, mrna, serum, proteinuria, hypertension, marker, flow cytometry, pe patient, serum level, onset, pregnant women, hypertensive disorder, gestational disorder, and the clinical syndrome. hypertensive disorder, gestational age, age, lymphocyte, diagnosis, risk, delivery, peripheral blood, and prevention. This group of topics examines the changes and mechanisms of immune cells that cause maternal clinical syndrome. **Figure 8B** presents those keywords according to the average time of appearance. We could observe that immune cell changes at the maternal-fetal interface, such as treg, were still the hot topic in recent years. **Figure 8C** indicated the evolution of the keywords studied over time and duration. In the last 3 years, the impact of pathways related to regulatory t-cells, th17, and vascular dysfunction on the development of HDP and the prediction of disease progression have been studied. Future research is likely to remain focused on investigating the specific mechanisms of different immune cells in the maternal-fetal interface, and the translation of related research.

### Related field analysis

**Figure 9:**
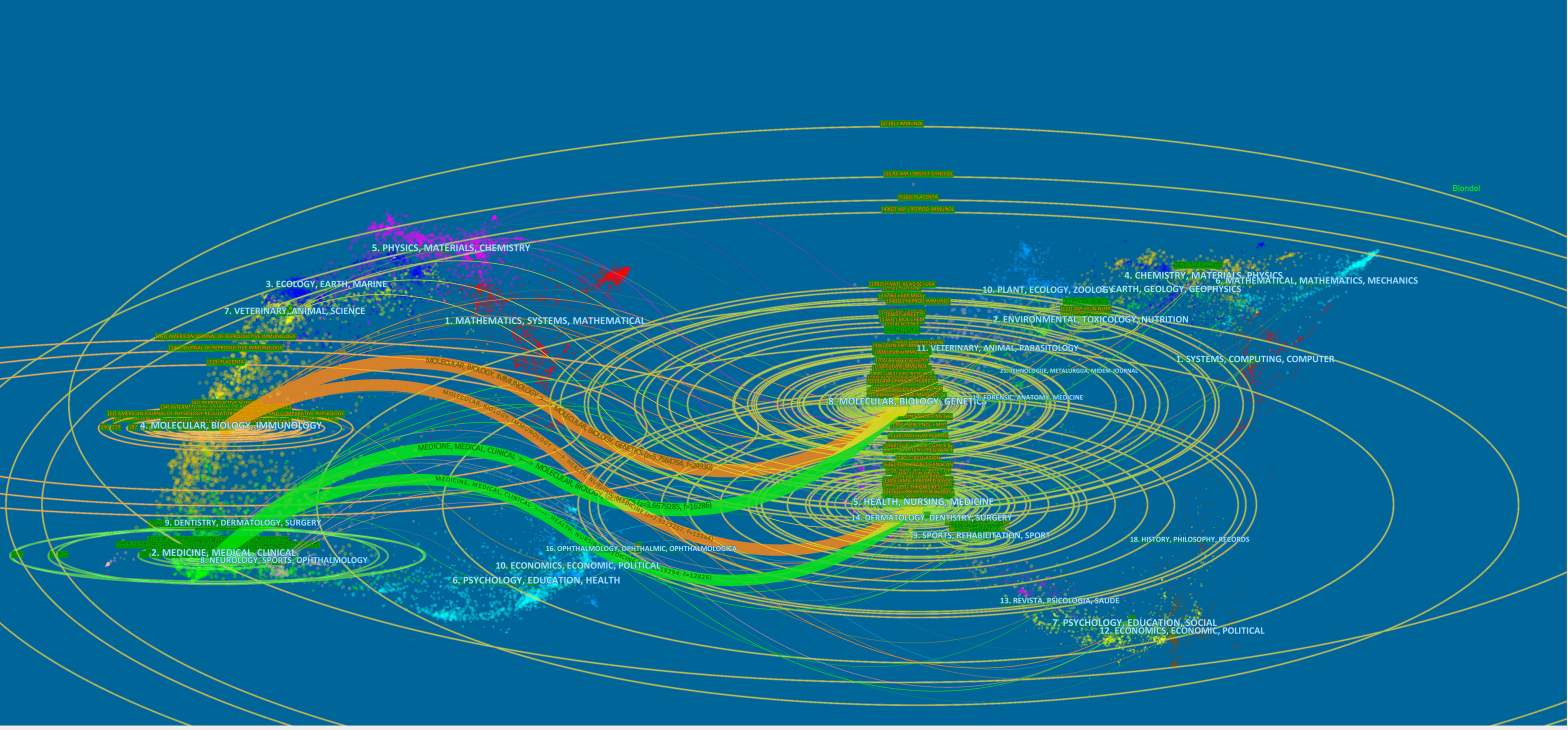
Fields related to HDP and immune cells.

The 2,388 publications we studied were divided into two main areas, one including molecular, biology, and immunology; the second area including medicine, medical, and clinical. In **Figure 9**, we could find that the references of these 2,388 articles were mainly distributed in the following fields: molecular, biology, immunology, genetic, medicine, medicine and clinical. In addition, nursing, sports also had some influence on this study. We found that the role of immune cells in HDP mainly involved molecular biological mechanisms related to genes, but we could not ignore the influence such as nursing and sports. The development of this field should not be limited to the in-depth exploration of molecular biology, but needs to consider multiple influences.

## Discussion

With the increasing number of the relevant studies, bibliometric analysis identifies data characteristics of research areas from the perspective of the literature, providing a clear and in-depth presentation of research content and trends in the form of knowledge graph (15, 16). In this study, we used bibliometric analysis to comprehensively summarize and visually analyze the records of published publications in the WoSCC for the past 20 years to identify research collaborations, trends, and hotspots in the field of HDP and immune cells research.

After excluding studies that did not meet the inclusion criteria by time, language, and article type, a total of 2,388 original articles and reviews were included in the bibliometric analysis, and these records included 2,388 English-language papers published in 593 journals from 2001 to 2021 by 2,174 universities/institutions in 91 countries/regions. The number of publications and citations in the field of HDP and immune cells research showed an overall upward trend over time, with a soaring trend in article citations since 2018. This indicates that the interest in HDP and immune cells research has surged in recent years and this area will remain a popular research in the coming years. The number of publications in a research area can reflect the level of scientific research in a country or institution (17). Based on the level of contribution of the countries/regions, the USA ranked first in the number of publications, with more than a quarter of the total number of included publications, indicating its influence in the field of HDP and immune cells research. In addition, the most influential universities/institutions were UNIVERSITY OF MISSISSIPPI (n=66)Finally, Lamarca B, Romero R, and Saito S were the top three authors in the number of publications, and Saito S was the top author in terms of citations, who might influence the focus and direction of research in this field. Collaboration is crucial to the research in this field, and we could find that the collaboration is widespread, with UNIVERSITY OF MISSISSIPPI as the largest initiating institution, which cooperated closely with several institutions. Fudan University, Nanjing Medical University and Wuhan University had produced more studies and were a active in international cooperation in the past three years. In the last 5 years, Lamarca B and Ibrahim T have made significant contributions to this research area through close collaboration among these authors.

From the bibliometric analysis we know all the relevant journals in the field, which can provide researchers with suitable options to retrieve publications or submit manuscripts (18). AMERICAN JOURNAL OF REPRODUCTIVE IMMUNOLOGY, JOURNAL OF REPRODUCTIVE IMMUNOLOGY and PLACENTA were the top three journals in the number of relevant articles published as well as in citations, and were the core journals publishing articles on HDP and immune cells. HYPERTENSION, JOURNAL OF REPRODUCTIVE IMMUNOLOGY and AMERICAN JOURNAL OF OBSTETRICS AND GYNECOLOGY were the journals with the highest IF among the top 10 journals by publications, and these journals might have published more potentially groundbreaking articles in the field that could provide some reference for researchers. The metrics of the top 10 journals by publications may provide information for scholars to submit their manuscripts (**Table 3**).

The mildest of HDP is gestational hypertension, chronic hypertension, followed by preeclampsia (either present after 20 weeks or superimposed on pre-existing chronic hypertension) and the most extreme form as eclampsia. Masked hypertension and white-coat hypertension also were also included based on a statement issued by the International Society for the Study of Hypertension in Pregnancy (ISSHP) in 2018 (1).Gestational hypertension, chronic hypertension, masked hypertension and white-coat hypertension have been found to have a tendency to progress towards preeclampsia, one of the major causes of the global maternal and perinatal morbidity and mortality. It has been shown that between 1990 and 2019, the number of people with HDP at the 204 countries/regions worldwide showed an increasing trend from 16.3 million to 18.08 million, with a 10.92% of increase in prevalence. Mortality and morbidity are on a decreasing trend in most countries/regions, with a 30.05% of reduction in mortality in 2019 compared to 1990 (19).Despite the current decrease in mortality, there is no effective treatment to improve maternal and infant outcomes, and thus, it necessitates a deep insight into into the pathogenesis of the disease in order to develop more effective treatment. Preeclampsia is a disease with multiple factors, mechanisms, and pathways, and multiple studies have attempted to understand the causes and mechanisms of preeclampsia development, with the widely accepted two stages of preeclampsia occurring - early gestational placental malformation followed by systemic endothelial dysfunction (a clinical syndrome secondary to uteroplacental malperfusion). The two processes of intravascular trophoblast invasion and helical artery remodeling during gestation in humans are dependent on proper regulation of the maternal immune system (20, 21). In early trimester, embryo implantation is a pro-inflammatory state, where pro-inflammatory cytokines released by immune cells as well as the endometrium itself promote tissue remodeling and angiogenesis to assist embryo implantation (22). However, pregnancy requires regulatory and anti-inflammatory cytokines released by immune cells such as Tregs to control pro-inflammatory cytokines and induce maternal immune tolerance to fetal antigens to ensure a healthy pregnancy (23). When there is any local imbalance in the immune response, it may cause superficial invasion of trophoblast cells in early gestation which may lead to impaired spiral artery remodeling and reduced placental vascular distribution, causing local ischemia and hypoxia in the placenta resulting in increased lipid peroxidation or decreased antioxidant activity, and this imbalance causes oxidative stress and inflammatory response, releasing multiple placental factors into the maternal circulation, inducing maternal systemic inflammatory reactions and vascular endothelial damage, causing clinical signs (24). Keyword co-occurrence analysis helps to capture and cluster current and future research trends, and we identified three clusters of HDP and immune cells research.

### Cluster 1: Immune cell changes during trophoblast invasion

Embryo implantation requires two parts: the “soil” - the decidualized endometrium, and the “seed” - the blastocyst. In recent years, many studies have found that the decidualized endometrium plays an important role in embryo invasion, spiral artery remodeling and the establishment of the maternal-fetal interface (25). The endometrium undergoes decidualization under hormonal, biochemical and immunological influences to prepare for pregnancy (26). During this process, endometrial stromal fibroblasts (ESC) differentiate into decidual stromal cells(DSC), a unique cell generated by ESC reprogramming in which many genes involved in the inflammatory response as well as resistance to invasion are decreased in expression and genes promoting cell proliferation are upregulated, which play an important part for embryonic implantation, invasion, and angiogenesisIn addition, DSC secretes several cytokines to promote the proliferation and differentiation of decidual natural killer cells (dNK) in early pregnancy. The degree of metaplasia is related to the depth of trophoblast invasion into the uterus. Therefore, proper decidualization is key to a healthy pregnancy (24). After embryo implantation, trophoblast cells proliferate rapidly to form anchoring villi anchored to the uterine wall, and cellular trophoblast cells from the anchoring villi implanted into extravillous trophoblasts (EVT). A portion of the EVT invade into the deeper layers of the decidua, and another portion of EVT invade the maternal uterine spiral artery, migrate along the inner lumen of the spiral artery, replace the maternal vascular endothelium, and transform the thick-walled uterine spiral artery into a high-volume and low-resistance uteroplacental artery vasculature. The process of trophoblast-mediated remodeling of the spiral arteries is essential to accomodate fetal placental blood flow to fetal development.In this period, NK cells, macrophages, T cells and dendritic cells (DC) in peripheral blood undergo a series of transformations and become decidual immune cells(DIC).These immune cells accumulate around trophoblast cells and can control the removal of cells from the spiral arteries, providing the necessary conditions for trophoblast cell migration (25).In addition, immune cells such as NK cells and macrophages in the metaplasia accumulate and secrete cytokines and chemokines that are crucial for promoting spiral artery transformation (27). In recent years, studies have concluded that abnormal alterations in the implantation stages determine susceptibility to preeclampsia. If trophoblast invasion into the decidua is defective, inadequate transformation of the uterine spiral arteries leads to abnormal development of the placenta, which is associated with pregnancy disorders such as pre-eclampsia and fetal growth restriction.When metaplasia is defective, imbalances in local immune response may affect trophoblastic invasion, angiogenesis and placental formation, resulting in incomplete remodeling of the spiral arteries and consequent placental insufficiency, which is associated with the pathogenesis of preeclampsia and fetal growth restriction. The resulting imbalanced immune response can also lead to placental apoptosis and necrosis (28). It was found that pregnant women with preeclampsia have a defect in decidua, which was associated with abnormal growth of the cellular trophectoderm, and this effect can persist for years afterpregnancy (29). In addition, it was found that conditioned medium from decidual cells of patients with severe preeclampsia failed to induce cytotrophoblast invasion (30). The development of preeclampsia is closely associated with decidualization and precedes gestation. It has been found to be associated with several pathways, which can be further investigated *in vitro* and intervened, allowing for new research directions for disease prevention.

### Cluster 2 immune tolerance at the maternal-fetal interface

The blastocyst is a mixture of maternal and paternal genomes, and When the maternal blood flow becomes active after placenta formation,the maternal immune system must accept the exposed semi-identical embryonic antigens and also resist infections or pathogens during pregnancy only in this way can the trophectoderm cells grow properly. When there is an immune imbalance, abnormal trophectoderm growth and differentiation can lead to placental insufficiency, which is associated with the pathogenesis of preeclampsia, miscarriage, and fetal growth restriction (31). In normal pregnancy, pro-inflammatory CD4+ T cell responses promote trophoblast invasion, placenta formation and anti-inflammatory responses, which in turn induce a shift in tolerance that is essential for embryonic development (32). This process involves multiple mechanisms, among which Treg, an important CD4+ T cell, transform the metaphase environment from a pro-inflammatory to an anti-inflammatory state and play an important role in maintaining the balance of the immune system and fetal immune tolerance during pregnancy (33). In contrast to the immunosuppression that occurs in normal pregnancy, preeclampsia is characterized by immune hyperactivation.It has been found that Tregs are reduced in maladaptive immune responses, and the degree of reduction in Tregs may be proportional to the severity of the disease (33). In addition, Human leukocyte antigen (HLA)-G have been suggested to be influential in inducing maternal immune tolerance to fetal antigens during pregnancy. HLA-G belongs to the non-classical Ib class genes that encode genes involved in spiral artery remodeling, immune tolerance, and fetal growth and development. In recent years, there has been an explosion of research on HLA-G biology, and current studies suggest that HLA-G is mainly selectively expressed by extravillous trophoblast cells at the maternal-fetal interface, and that HLA-G interacts with NK cells to promote spiral artery remodeling (34). Moreover, it can inhibit the maternal immune response to fetal tissues, which in turn serves to protect the embryo (35). In addition, HLA-G has been found to stimulate the secretion of growth-promoting factor (GPF) in NK cells to accelerate fetal growth (36). Abnormal expression and genetic polymorphisms of HLA-G have been found to correlate closely with preeclampsia and recurrent miscarriage (34). A special microenvironment is formed at the maternal-fetal interface, and changes in immune cells and abnormal expression of HLA-G within this interface promote the disease development and can be further explored in future studies.

### Cluster 3 maternal clinical syndrome

Preeclampsia,as a hypertensive disorder of pregnancy, is one of the serious complications endangering the safety of mother and infant, characterized by systemic immune activation affecting the function and metabolism of several organs, and is featured by the development of a clinical syndrome manifested by hypertension with proteinuria (24h urine protein quantification≥0.3g or random urine protein≥2+) or without proteinuria when combined with systemic organ damage, such as thrombocytopenia, liver function impairment, renal insufficiency, pulmonary edema, or brain or visual impairment (5). As research progresses, it is increasingly recognized that there are multiple subtypes of preeclampsia, which are now divided into early-onset preeclampsia (EOPE) and late-onset preeclampsia (LOPE), using 34 weeks of gestational age as the cut-off.EOPE is more closely associated with intrinsic placental factors such as impaired trophoblastic invasion, spiral artery remodeling, placental ischemia and hypoxia, and inflammatory damage (37), whereas LOPE is more correlated with placental overgrowth, cellular senescence, and maternal susceptibility factors such as age, lifestyle (24). Although the etiology of preeclampsia is currently controversial, it is now widely accepted that preeclampsia is a state of oxidative stress (38). In healthy pregnancies, oxidative stress occurs in the placenta with the advent of the hyperoxic environment, it can be regulated by increased antioxidant activity. Oxidative stress in the placenta caused by an imbalance between reactive oxygen species (ROS) production in cells and tissues and antioxidant capacity triggers the release of pro-inflammatory cytokines, apoptotic debris and angiogenic factors to the maternal circulation, which can induce an inflammatory response to maternal endothelial dysfunction and facilitate the development of preeclampsia (39). Increased levels of oxidative stress markers such as 4-hydroxynonenal (HNE) and malondialdehyde (MDA) in maternal peripheral blood and placenta and a decrease in antioxidant activity were found in patients with preeclampsia (40). It is now widely recognized that placental ischemia promotes the release of pro-inflammatory cytokines such as TNF-α, IFN-γ and certain interleukins (e.g., IL-2, IL-6, etc.) from the placenta into the maternal circulation, and these cytokines then lead to increased expression of adhesion molecules and endothelial dysfunction in the maternal vascular system, which is positively associated with increased blood pressure, and this contributes to the pathogenesis of preeclampsia (41). TNF-α induces the release of several inflammatory and growth cytokines, which may affect maternal immune cells by altering the secretion of immunomodulatory factors from the placenta. Elevated levels of TNF-α have been found to be associated with the development of HDP (42). High levels of IFN-γ secretion have been reported to persist in the placenta and maternal plasma of patients with preeclampsia. However, further studies have shown that IFN-γ was required during embryo implantation and that IFN-γ levels above or below the threshold may be harmful to the pregnant woman. In addition, IFN-γ could reverse the effects of TNF-α on EVT during metaphase invasion (43).There are no sensitive serum biochemical markers to predict preeclampsia. Current studies have found significant vascular damage at all stages of preeclampsia, from the onset of placentation to the postpartum period. Preventing immune imbalance at an early stage may reduce the occurrence of adverse placental formation and oxidative stress, and how to prevent preeclampsia before the onset of symptoms needs further study.

In summary, we can see that the development of HDP is closely related to alterations in maternal innate and adaptive immunity. The most distinctive feature of pregnancy is the ability of the mother to develop immune tolerance to the “semi-allogeneic fetus” and meantime to maintain a strong immune response to pathogenic infections. It is now believed that immune factors are involved in all phases of the development of preeclampsia, and that an imbalance between pro- and anti-inflammatory events during pregnancy can result in a chronic inflammatory response and consequent phenotypic changes in HDP. The largest fixed immune cell population in the decidua endometrium is lymphocytes, and there is great heterogeneity in immune cell subsets at different times of pregnancy. In early pregnancy, dNK cells are the most abundant cell subset, account for approximately 70% of the leukocytes in the decidua (44), followed by macrophages (approximately 20%), T cells (approximately 10%), and only approximately 1% are dendritic cells. As pregnancy continues, dNK cells gradually decrease, macrophage percentage remains relatively constant, and T cells gradually increase (45). The correlation between the development of pre-eclampsia and imbalance of immunity in the placental and maternal circulation has been widely described in the last two decades. Numerous studies have shown that quantitative and qualitative alterations in the maternal system and local immune cells induce the entry of multiple chemokines, pro-inflammatory cytokines and anti-angiogenic factors into the maternal circulation, which lead to the systemic effects of pre-eclampsia. With the changing keywords in the focus of research on the disease, we found that the main focus of research on preeclampsia has been on the following areas:

NK cells: they are triggered by the expression of specific receptors interacting with ligands and are mainly activated by the release of cytotoxic and pro-inflammatory cytokines (46).NK cells are mainly classified into peripheral natural killer cells (pNK), uterine natural killer cells (uNK), decidual natural killer cells (dNK) based on the location of NK cells and their surface markers. uNK cells have a different phenotype from peripheral pNK cells and have a strong secretory capacity. uNK cells are currently thought to have several possible sources and can differentiate into dNK cells by interacting with the metaphase microenvironment (47). dNK cells are the most important lymphocytes in the metaphase of early and mid pregnancy. They account for about 70% of the total lymphocytes in these pregnancies and about 50% in late pregnancy (48). Studies have shown that dNK cells play an important role in uterine spiral artery remodeling, placental development, and resistance to microbial infections. In normal pregnancy, the maternal-fetal interface is immune tolerant and activated dNK cells play an important role in defending against infection and preventing intra-placental transmission in the presence of pathogenic microorganisms (47). dNK cells are recruited in large numbers near the uterine spiral arteries and interact with ligands on the surface of trophoblast cells to activate dNK cells to secrete cytokines and angiogenic factors, induce de-differentiation of spiral arterial vascular smooth muscle cells (49), as well as promote the recruitment and invasive capacity of EVT cells (50). When there is an immune imbalance, abnormal activation of NK cells triggers HDP pathological changes. It was found that endometrial and dNK cell maturation is impaired during the secretory phase of the endometrium and during early pregnancy in women who develop preeclampsia (51). In addition, it has been proposed that excessive levels of cytokines, such as TGF-β, can mediate excessive activation of dNK cells, affecting uterine spiral artery remodeling and contributing to the development of pre-eclampsia (52). It has been proposed that the killer immunoglobulin-like receptor (KIR), expressed on NK cells, is a key regulator of NK cell function and modulates NK cell activity by recognizing with specific human leukocyte antigen (HLA) molecules in the fetus, affecting maternal immune tolerance in the fetus, which influences the development of HDP (53). It was found that the absolute number of KIR ligand mismatches is significantly elevated in HDP and the homeostatic activation of NK cells affects the invasion of trophoblast cells into the uterine spiral arteries, leading to inadequate placental perfusion, so that KIR receptor-based diagnostics can be detected and used as a predictive HDP risk marker (54). However, studies on the specific regulation of the number, phenotype and function of dNK cells involved in the development of HDP are still at the stage of inference rather than evidence, further validation studies are still needed.

T cells: T cells represent the adaptive immune response and can be subdivided into CD8+ T fine and CD4+ T cells depending on their surface molecules and mode of action. Initial CD4+ T lymphocytes receive antigenic stimulation and produce different types of cytokines that can differentiate into effector T cells (including Th1, Th2, Th17, etc.) and Treg (55). Healthy pregnancies were thought to be driven by CD4+ T lymphocyte cells bias. Numerous studies have shown that immune imbalance mediated by CD4+ T lymphocytes cells and their associated immune products is a key factor in the development of preeclampsia. During normal pregnancy, Th1/Th2 and Treg/Th17 cells have a fine dynamic balance between Th1/Th2 function in favor of Th2 and Treg/Th17 function in favor of Treg, which ensures protection of the embryo from attack while avoiding infection. However, the balance between Th1/Th2 functions in peripheral blood and decidua changes toward a Th1-dominated inflammatory phenotype in preeclamptic patients (56, 57). Th1 induced chronic inflammatory reactions in the fetal- maternal interface, placental damage and endothelial dysfunction by secreting the amounts of TNF-α, IFN-γ, IL- 1β, and IL- 12 (58).Preeclamptic symptoms such as elevated blood pressure, proteinuria and renal function abnormalities occur in pregnant mice after Th1 cell importation (59). A growing number of studies have found that disruption of Treg/Th17 balance can lead to the development of preeclampsia and that the balance between Treg and Th17 is essential for the prevention of preeclampsia. It was found that in preeclampsia, a decrease in Treg in maternal peripheral blood and tissue was accompanied by an increase in pro-inflammatory Th17, CD8+ effector cells and trophoblast apoptosis (33).Th17 Induced cytolytic NK cells’ function, placenta perforin production, IL-17, IL-6, IFN-γ, and TNF-α cytokines. It was found that Th17 in normal pregnant rats induced activation of NK cells, increased plasma TNF-α levels, and decreased placental VEGF, ultimately leading to preeclampsia-related symptoms (60). In addition, it was found that PD- 1/PD-L1 pathway disorders can affect the Treg/Th17 balance in peripheral blood and decidua during pregnancy, leading to the development of preeclampsia (61). However,the feasibility of the treatment options needs to be further validated.

Monocytes/macrophages: Monocytes/macrophages are important cellular components of the human innate immune system, including monocytes in the circulatory system and macrophages in tissues. The maternal immune response is manifested by altered functional activity of the monocyte-macrophage system with the development of preeclampsia. It was found that monocyte counts and ratios in the circulatory system were significantly elevated in patients with preeclampsia (62, 63), which led to the suggestion that quantitative and qualitative characteristics of monocytes in the circulatory system reflect the severity of preeclamptic pregnancy. In recent years, an increasing number of studies have focused on the changing characteristics of monocyte subpopulations. Currently, monocytes are divided into three main categories: classical monocytes, non-classical monocytes, and intermediate monocytes. It was found that in patients with pre-eclampsia, the level of anti-inflammatory classical monocytes decreased, while the level of pro-inflammatory intermediate monocytes and non-classical monocytes showed a significant increase (64, 65). When pro-inflammatory monocytes are increased in patients with preeclampsia, the secreted cytokines make the systemic inflammatory response also enhanced (66). However, the specific mechanism of monocyte activation during pregnancy is unclear, and it has been hypothesized that the placenta plays a dominant role, with monocytes activating the proinflammatory phenotype through placental contact with the syncytial trophoblast (67), in addition to indirect activation of monocytes by cytokines released into the blood, microvesicles, exosomes and pregnancy hormones (68). The current study found that blood monocyte counts can be a predictor of disease progression in preeclampsia, but a prospective study is needed to evaluate the predictive role of monocytes in the blood of pregnant women with preeclampsia.

Macrophages, mainly derived from monocytes in the circulatory system, exceed 20%-30% of the total number of macrophages in the body and secrete cytokines and growth factors involved in uterine spiral artery remodeling, in addition to providing immune tolerance to the fetus and protecting the fetus from infection (68). Macrophages in tissues can be divided into two categories: pro-inflammatory (M1) macrophages and anti-inflammatory (M2) macrophages. In different environments, macrophages are able to differentiate to specific functional phenotypes of pro-inflammatory (M1) or anti-inflammatory (M2). The balance between pro-inflammatory (M1) and anti-inflammatory (M2) macrophages in the placenta is essential for a normal pregnancy. It has been suggested that in mid-pregnancy, macrophages differentiate into M2 macrophages, however, this process is inhibited in pre-eclamptic pregnancies, while M1 macrophages are unaffected, showing high levels of pro-inflammatory cytokines, such as IFN-γ, TNF-a, IL6, and low levels of anti-inflammatory cytokines, such as IL-4 and IL-10 (69). In addition to cytokines, cytosolic axis and placental mesenchymal stem cells also have an effect on macrophage differentiation, however, the specific behavioral changes of macrophages in pre-eclampsia regarding when the altered macrophage differentiation occurs still need to be further explored by investigators.

Other: Dendritic cells (DCs) in the placenta are relatively few in number and show low proliferative capacity, and DCs have been found to regulate Th2 to maintain immune tolerance; however, the specific role of DCs in the pathogenesis of HDP is unknown (70). Placenta-derived particles can effectively activate neutrophils to produce neutrophil extracellular traps (NETs) *in vitro* (71). NETs have been reported to promote endothelial cell activation *via* IL-1a and histone G, which in turn leads to increased thrombosis (72). However, it is not clear whether neutrophil infiltration also leads to endothelial cell activation in HDP. The specific functions of other immune cells need to be further explored.

### The future research directions

A retrospective review of the findings of the last 20 years has mostly focused on quantitative and qualitative changes of immune cells in the placenta and circulation in HDP, corresponding to secreted chemokines, pro-inflammatory cytokines and anti-angiogenic factors,and the mechanisms of interaction. Throug the high quality articles published in recent years, we found an increasing number of researchers referring to predictive markers, immunotherapies and potential interventions.

First, the influence of platelets on the development of HDP, its predictive ability and symptomatic treatment remain research hotspots based on the changing hotspots of keyword research. It is now believed that human platelets are also immune cells, interacting with the complement system and other immune cells to activate the immune system and trigger the development of related diseases (73). In recent years, studies on platelets and HDP have gradually increased. It was found that platelet activation triggers a complex signaling cascade response in which receptors in the platelet cytoplasm are relocated to the platelet surface, facilitating interactions with immune cells, such as monocytes, macrophages and endothelial cells. Thus, platelet activation triggers a local inflammatory response that releases chemokines, upregulates surface receptors and increases intercellular interactions. Platelet activation causes alterations in endothelial cell phenotype, increased leukocyte recruitment and an inflammatory response at the maternal-fetal interface, resulting in placental alterations and an increased risk of adverse pregnancy outcome (74). A recent study found that platelets activate a large release of TGF-β, which is also a possible mechanism of platelet-trophoblast interaction (75). Platelet alterations have been suggested as predictors of HDP disease progression, however, studies on the specific regulation of platelet involvement in the development of preeclampsia are still need to be further explored in the future. The second is about the development of immunotherapies. It is well known that excessive inflammatory response is a key driver of preeclampsia, and alterations in the number and activity of immune cells may prevent or inhibit the development of the disease, for example, researchers have now proposed CAR T-cell therapy to ameliorate the development of preeclampsia from theory (76), however, for the exact corellation between impaired immune cells and preeclampsia, the underlying mechanism, the validation of preclinical rodent models, and the feasibility of immunotherapies still need to be further explored and validated. Finally, we still need to explore markers with high sensitivity and specificity and the exact mechanism, which are important for the future exploration of the optimal timing of intervention, prevention and development of therapeutic approaches for HDP.

Although we conducted a relatively comprehensive analysis of HDP and immune cells research from 2001-2021. some limitations are inevitable. First, as for the inclusion criteria, we limited time, language, and article type, and some article types published in other formats such as non-English, conference abstracts, and the most recent studies in 2022 were excluded, which may lead to neglect of some influential articles. Second, the database is still dynamically updated, and some articles may not have had enough time to be discovered or cited by scholars in related fields, which reflects time-lagged nature of bibliometric analysis; finally, we searched the core database from WOSCC and ignored articles in related fields from non-core or other search engines, which may also result in our study not being exhaustive. However, the aim of this study was to conduct a high-quality bibliometric analysis of HDP and immune cells research; therefore, these limitations did not affect the current findings.

## Conclusions

This study provides a comprehensive bibliometric analysis of HDP and immune cells from 2001 to 2021, revealing the major research countries/regions, universities/institutions and authors, historical evolution as well as the current research status and future research hotspots. The results showed an upward trend in HDP and immune cells research. Keywords were organized into three clusters, including immune cell changes during trophoblast invasion, immune tolerance at the maternal-fetal interface, and maternal clinical syndrome. The study concerning the specific mechanisms and the current research status of different immune cells in the maternal-fetal interface on the progression of HDP and future scientific prospects.Platelets correlate with HDP and translational research for the prophylaxis or treatment of HDP remain priorities for the future research.

## Data availability statement

The datasets presented in this study can be found in online repositories. The names of the repository/repositories and accession number(s) can be found in the article/**Supplementary Material**.

## Author contributions

FT conceived the study and critically revised the content of this manuscript. YW and BL made significant contributions to the study methods, results, and writing of the manuscript. All authors contributed to the article and approved the submitted version.
