# Supplementary material for: Global trends in research of immune cells associated with hypertensive disorders of pregnancy: A 20-year bibliometric analyses (from 2001 to 2021)
Source: Front Immunol. 2023 Jan 9;13:1036461. doi: 10.3389/fimmu.2022.1036461 (PMC9868159; doi:10.3389/fimmu.2022.1036461)
Supplement: Supplementary file 1 [file Table_1.docx]

### [Supplementary Material S1](https://www.ncbi.nlm.nih.gov/pmc/articles/PMC8934933/#SM3) Historiograph of HDP and immune cells research.

| Rank | Title | Journal | Year | LCS | GCS |
| --- | --- | --- | --- | --- | --- |
| 1 | Macrophage-induced apoptosis limits endovascular trophoblast invasion in the uterine wall of preeclamptic women | LAB INVEST | 2001 | 66 | 204 |
| 2 | Endovascular trophoblast invasion: Implications for the pathogenesis of intrauterine growth retardation and preeclampsia | BIOL REPROD | 2003 | 71 | 789 |
| 3 | Th1/Th2 balance in preeclampsia | J REPROD IMMUNOL | 2003 | 101 | 223 |
| 4 | Combinations of maternal kir and fetal hla-c genes influence the risk of preeclampsia and reproductive success | J EXP MED | 2004 | 185 | 775 |
| 5 | Changes in systemic type 1 and type 2 immunity in normal pregnancy and pre-eclampsia may be mediated by natural killer cells | EUR J IMMUNOL | 2005 | 76 | 159 |
| 6 | The uterine spiral arteries in human pregnancy: Facts and controversies | PLACENTA | 2006 | 113 | 731 |
| 7 | Cytokine mapping of sera from women with preeclampsia and normal pregnancies | J Reprod Immunol | 2006 | 72 | 188 |
| 8 | Systemic inflammatory priming in normal pregnancy and preeclampsia: The role of circulating syncytiotrophoblast microparticles | J IMMUNOL | 2007 | 80 | 312 |
| 9 | Proportion of peripheral blood and decidual cd4(+) cd25(Bright) regulatory t cells in pre-eclampsia | CLIN EXP IMMUNOL | 2007 | 133 | 243 |
| 10 | Preeclampsia is associated with lower percentages of regulatory t cells in maternal blood | HYPERTENS PREGNANCY | 2009 | 74 | 109 |
| 11 | Altered decidual leucocyte populations in the placental bed in pre-eclampsia and foetal growth restriction: A comparison with late normal pregnancy | REPRODUCTION | 2009 | 62 | 102 |
| 12 | Evidence for immune cell involvement in decidual spiral arteriole remodeling in early human pregnancy | AM J PATHOL | 2009 | 104 | 280 |
| 13 | Systemic increase in the ratio between foxp3(+) and il-17-producing cd4(+) t cells in healthy pregnancy but not in preeclampsia | J IMMUNOL | 2009 | 149 | 315 |
| 14 | Maternal activating kirs protect against human reproductive failure mediated by fetal hla-c2 | J CLIN INVEST | 2010 | 70 | 316 |
| 15 | Immunology of pre-eclampsia | AM J REPROD IMMUNOL | 2010 | 118 | 443 |
| 16 | Th1/Th2/Th17 and regulatory t-cell paradigm in pregnancy | AM J REPROD IMMUNOL | 2010 | 124 | 712 |
| 17 | Circulating cytokines, chemokines and adhesion molecules in normal pregnancy and preeclampsia determined by multiplex suspension array | BMC Immunol | 2010 | 83 | 340 |
| 18 | Increased prevalence of IL-17-producing peripheral blood lymphocytes in pre-eclampsia | AM J REPROD IMMUNOL | 2011 | 67 | 115 |
| 19 | The predominance of th17 lymphocytes and decreased number and function of treg cells in preeclampsia | J REPROD IMMUNOL | 2012 | 106 | 166 |
| 20 | Uterine natural killer cells initiate spiral artery remodeling in human pregnancy | FASEB J | 2012 | 63 | 196 |

GCS, the total number of citations in Web of Science; LCS, the number of citations in the current dataset.
